# Gut microbial metabolites SCFAs and chronic kidney disease

**DOI:** 10.1186/s12967-024-04974-6

**Published:** 2024-02-18

**Authors:** Meng He, Wenqian Wei, Yichen Zhang, Zhouxia Xiang, Dan Peng, Ayijiaken Kasimumali, Shu Rong

**Affiliations:** 1 Department of Nephrology, Shanghai General Hospital, Shanghai Jiao Tong University School of Medicine, Shanghai, 200080 China; 2 Department of Urology, Shanghai General Hospital, Shanghai Jiao Tong University School of Medicine, Shanghai, 200080 China

**Keywords:** SCFAs, CKD, Gut microbial metabolites, Inflammatory responses, Oxidative stress

## Abstract

The global incidence of Chronic Kidney Disease (CKD) is steadily escalating, with discernible linkage to the intricate terrain of intestinal microecology. The intestinal microbiota orchestrates a dynamic equilibrium in the organism, metabolizing dietary-derived compounds, a process which profoundly impacts human health. Among these compounds, short-chain fatty acids (SCFAs), which result from microbial metabolic processes, play a versatile role in influencing host energy homeostasis, immune function, and intermicrobial signaling, etc. SCFAs emerge as pivotal risk factors influencing CKD’s development and prognosis. This paper review elucidates the impact of gut microbial metabolites, specifically SCFAs, on CKD, highlighting their role in modulating host inflammatory responses, oxidative stress, cellular autophagy, the immune milieu, and signaling cascades. An in-depth comprehension of the interplay between SCFAs and kidney disease pathogenesis may pave the way for their utilization as biomarkers for CKD progression and prognosis or as novel adjunctive therapeutic strategies.

## Background

The human intestinal tract harbors an abundant population of numerous microorganisms, encompassing a multitude of microbial categories, including probiotics, neutrophils, and pathogens, that coexist and engage in dynamic competition within the gut ecosystem [1]. These intricate microbial communities collectively function as a 'second genome', wielding a profound influence over the host’s metabolic pathways, and thereby governing the intricate homeostatic equilibrium of the body's physiology [2]. Consequently, research on the intestinal flora has garnered progressive attention within the academic community. As early as 2005, the French National Institute of Agricultural Research (INRA) introduced the Human Intestinal Metagenome Initiative (HIMI), while the 'Human Microbiome Project (HMP)' initiated by the National Institutes of Health (NIH), was subsequently launched to undertake an extensive investigation into the crucial role of the gut microbiome in human health and disease [3].

The global incidence of kidney diseases is in an escalating trajectory. As of a 2017 survey, the worldwide prevalence of CKD surpassed 697 million cases, accounting for a prevalence rate of 9.1%. Notably, CKD-related mortality has experienced an alarming surge, registered over 1.2 million deaths and reflecting a substantial 41.5% escalation in all-age mortality when juxtaposed with rates from 1990 [4]. Projections indicate that by 2040, CKD is on the brink of becoming the fifth most prominent contributor to worldwide mortality [5]. Presently, the management of CKD primarily focuses on retarding disease progression and safeguarding renal function. In recent years, comprehensive gene sequencing has substantiated the intimate association between the gut microbiota and its metabolic byproducts and the initiation and advancement of CKD [6]. Consequently, elucidating the nexus between gut flora and CKD holds the promise of unveiling novel research horizons for future kidney disease management, and potentially ushering in a novel therapeutic avenue for the utilization of SCFAs in CKD treatment.

The relationship between the human body and intestinal flora is primarily mediated by intestinal flora-produced metabolites. Alterations in the intestinal flora have a discernible impact on the trajectory of kidney disease by modulating the production of these intestinal metabolites. Evidence supports a correlation between SCFAs and a range of kidney disorders, including CKD, end-stage renal disease (ESRD), and diabetic nephropathy [7]. Despite the recognized association between SCFAs and kidney disease, a comprehensive elucidation of the mechanisms through which SCFAs govern disease development and progression remains elusive. This paper seeks to delve into the involvement of SCFAs in the pathophysiology of CKD and to assess the potential for targeted therapeutic interventions, with a focus on reviewing the influence of dietary regimens and the regulation of gut flora on SCFA modulation. Thus, our aim is to unravel the intricate mechanisms underpinning SCFAs’ role in CKD development and to proffer innovative approaches to tackle this disease.

## Gut microbiota and short-chain fatty acids

Difficult-to-digest carbohydrates in food, including dietary fibre, resistant starch and oligosaccharides, are subject to fermentation by anaerobic colonic bacteria, resulting in the generation of SCFA. Comprising nearly 95% of SCFAs are acetate, propionate, and butyrate [8]. After their production in the colon, only a small portion is present in the intestine in the unionized form that can cross the epithelial barrier directly, whereas approximately 80–90% of short-chain fatty acids are present in an ionized form and require specific transporters for their absorption [9]. The transport of these SCFAs into the bloodstream is facilitated by several mechanisms. These include active transport mediated by hydrogen ion-coupled monocarboxylate transporter (MCT)-1 and MCT-4, which are present on the parietal and basolateral membranes of the colon epithelium, as well as sodium-monocarboxylate transporter (SMCT)-1 and SMCT-2, found on the parietal membrane of the colon epithelium [10]. Additionally, direct diffusion through the cell membranes of the luminal cells plays a role in mediating SCFAs’ entry from the lumen into the bloodstream [11]. Following their generation in the colon, acetate and propionate flow into the bloodstream, where they travel through the portal vein to reach the liver. Here, SCFAs can serve as tricarboxylic acid cycle substrates, generating energy and participating in glucose synthesis. Notably, propionate is metabolized within hepatocytes. A portion of the acetate remains in the liver, while another part enters the systemic circulation through the peripheral vascular system. In contrast, butyrate, primarily serving as an energy source for colonic epithelial cells, is produced within the colon and utilized locally [12]. Consequently, only acetate is discernible in the peripheral bloodstream. While propionate and butyrate levels are notably low in peripheral blood, their presence can still exert a substantial influence on the operation of peripheral organs and tissues [1]. SCFAs exert their biological roles mainly as G protein-coupled receptors (GPCRs) GPR41 (Free Fatty Acid Receptor 3 FFAR3), GPR43 (Free Fatty Acid Receptor 4 FFAR4), GPR109A (Hydroxycarboxylic Acid receptor 2, HCAR 2) and ligands for the olfactory receptor (Olfr78) or inhibitors of histone deacetylases (HDACs). It has also been found that SCFAs, mainly butyrate, can also modulate the body's immune function by interacting with the aryl hydrocarbon receptor (AHR) [13], whereas Alex [14] confirmed the ability of propionate and butyrate to stimulate angiopoietin-like protein 4 (ANGPTL) by interacting with peroxisome proliferator-activated receptor c (PPARc) on colorectal adenocarcinoma cells synthesis, As shown in Table 1.

**Table 1:** Identified receptors for short-chain fatty acids

Receptor	Ligand	Expression	Function	References
GPR41 (FFAR3)	Acetate Propionate Butyrate	Kidney; Spleen; Bone marrow; Lymph nodes; Adipocytes; Pancreatic beta cells; Enteroendocrine cells; Vascular endothelial cells; Peripheral nervous system cells, etc	Inhibits cellular proliferation and provokes apoptotic responses; Regulates energy homeostasis; Modulates T-cell differentiation and immune responses; Facilitates sympathetic activation and triggers the release of the anorexigenic gut hormone, peptide YY (PYY)	[15–19]
GPR43 (FFAR2)	Acetate Propionate Butyrate	Adipocytes; Eosinophils; Basophils; Neutrophils; Immune cells, etc	Lipid metabolism; Energy homeostasis; Immune response; Neutrophil migration; Inhibition of fat accumulation; Secretion of enteric prohormones GLP-1, insulin and IgA	[20, 21]
GPR109A (HCAR2)	Butyrate	Intestinal epithelial cells; Adipocytes; Neutrophils; Macrophages; Monocytes; Dendritic cells, etc	Organismal metabolism; Cancer; Immunomodulation	[22]
Olfr78 (Olfactory receptor)	Acetate Propionate	Vascular smooth muscle cells	Control of blood pressure; Automatic regulation of tissue blood flow	[23–25]
HDACi	Propionate Butyrate	Histone	Anti-tumour; Anti-fibrotic; anti-inflammatory properties; Regulates the expression of immunomodulatory genes	[26, 27]
AHR	Butyrate	Treg; Dendritic cells; Stem cells	Modulation of immunity	[13]
PPARc	Propionate Butyrate	Adenocarcinoma cells of the colon	Stimulation of angiopoietin-like protein 4 (ANGPTL 4) synthesis	[14]

**Fig. 1:**
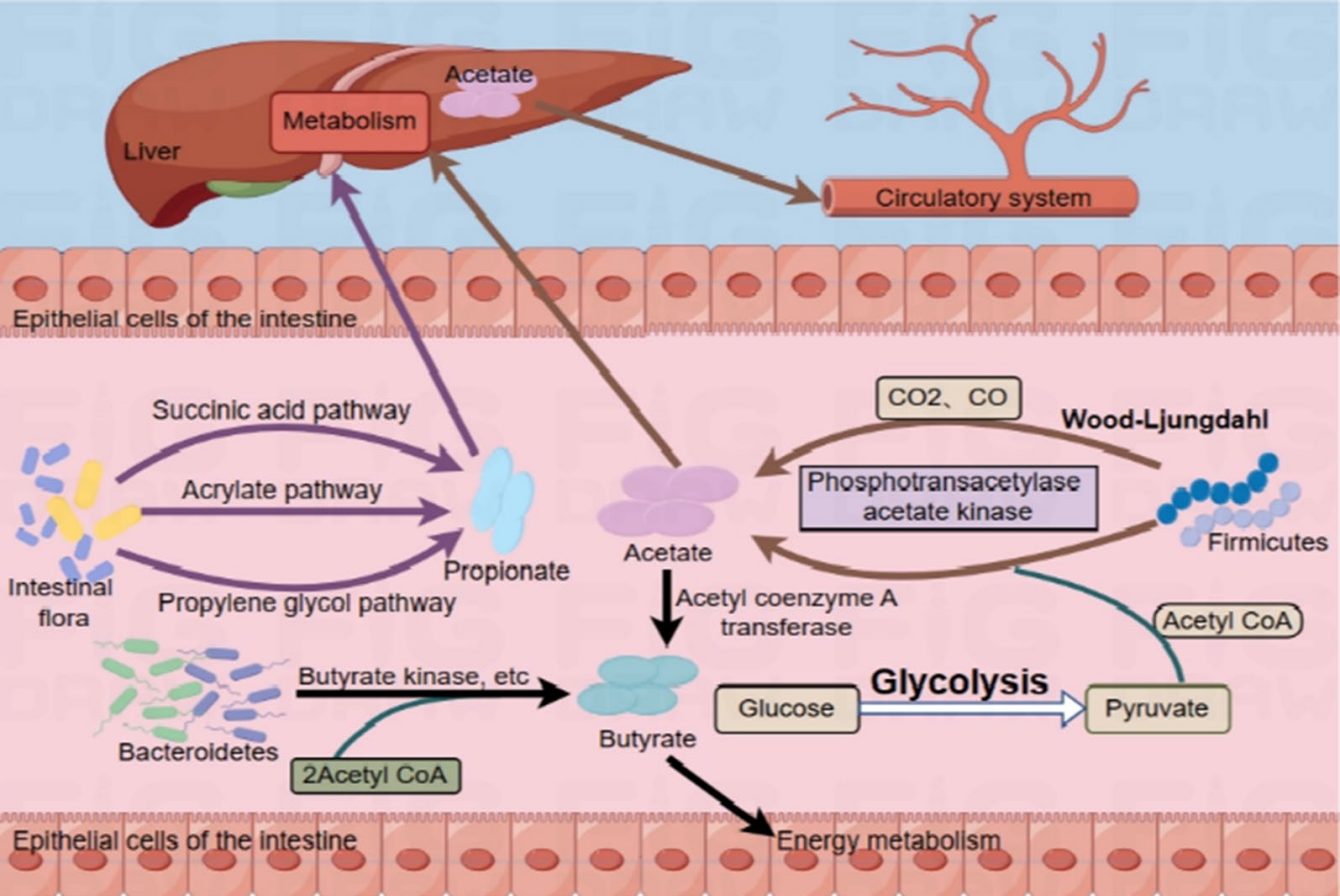
Synthesis and metabolism of SCFAs in the gastrointestinal tract. In the gut, the intestinal microbial metabolites of SCFAs, acetate, propionate and butyrate, are synthesised through different pathways under the action of different flora and are involved in modulating various metabolic processes within the organism

SCFAs can be produced by intestinal flora through different metabolic pathways, and the specific mechanisms are shown in Fig. 1 [28–31]. The glycolytic pathway is the predominant metabolic pathway for the production of SCFAs, whereby monosaccharides hydrolyzed by microorganisms in the colon are fermented to phosphoenolpyruvic acid via the Embden Meyerof pathway, and then phosphoenolpyruvic acid intermediates are used to produce SCFAs through different reactions [32]. The production of acetate relies on two main pathways: most notably, the *Firmicutes* utilize CO_2_, CO via the Wood-Ljungdahl pathway, which is also considered to be the most efficient production pathway for the production of acetate [31]. Additionally, pyruvate produced during glycolysis can also produce acetate via acetyl coenzyme A. The production of acetate via both the Wood-Ljungdahl pathway and the glycolytic pathway requires the catalytic action of phosphotransacetylase and acetate kinase [29]. Propionate can be produced via the succinate, acrylate, or propylene glycol pathways, and butyrate is usually produced by the condensation of two acetyl coenzyme A molecules by butyrate kinase or butyl coenzyme A: acetate coenzyme A transferase by the *cytophilic* and *xanthobacterial* groups of the phylum *Mycobacterium anisoplakii* [30] or by the conversion of butyrate via acetyl coenzyme A transferase using acetate as a substrate. An additional subset of bacteria, such as *Bifidobacteria*, can also produce the same metabolite SCFAs via the pentose phosphate pathway [28].

## SCFAs and CKD

CKD has emerged as a escalating worldwide public health concern, with rising morbidity and mortality rates year after year [33]. According to Kidney Disease: Improving Global Outcomes (KDIGO), CKD is defined as structural or functional renal abnormalities of ≥ 3 months’ duration due to various causes, including pathological injuries with normal and abnormal glomerular filtration rate (GFR), abnormalities in blood or urine composition and abnormalities in imaging, or unexplained decrease in glomerular filtration rate (GFR < mL/min.1.73 m^2^) for more than 3 months [34]. In contrast, patients with CKD stage 5 have a decrease in GFR to less than 15 mL/min.1.73 m^2^, at which point renal replacement therapy is often required. The main causes of CKD include chronic glomerular disease, type 2 diabetes mellitus, hypertension, obstructive nephropathy, cystic renal lesions, and rheumatologic immune system diseases such as systemic lupus erythematosus. In western countries such as the United States and the United Kingdom, diabetes mellitus and hypertension are the predominant etiologic factors [35], whereas in China, chronic glomerular disease is the most common cause of CKD, but the incidence of diabetes mellitus and hypertension leading to CKD is increasing year by year with the change of dietary structure and pattern. Worldwide, diabetes accounts for 30–50% of the causes of CKD, affecting approximately 285 million adults [36]. The yearly increase in the prevalence of type 2 diabetes, hypertension, obesity, and population aging in China will increase the burden of CKD in China. CKD is often complicated by cardiovascular disease, abnormal mineral metabolism bone disease, disorders of water-electrolyte and acid–base balance, anemia, and other disorders, of which cardiovascular disease is one of the leading causes of death in patients with CKD. Studies have demonstrated a significant reduction in the population of SCFAs-producing bacteria in the gut of individuals in CKD stage 5. This decrease in flora abundance is further accentuated as renal function declines, and the extent of reduction in flora abundance exhibits a positive correlation with the progression of the disease [37]. In 2014, VaziriND supplementation of CKD rats with high rectilinear resistant starch ameliorated oxidative stress, inflammatory state, and delayed the process of CKD in CKD rats [38]. The physiological roles of SCFAs in decelerating the advancement of CKD are being progressively elucidated, including the inhibition of inflammatory responses, suppression of oxidative stress, modulation of autophagy, and improved energy metabolism, and immune pathways.

### Inhibition of inflammatory responses

**Fig. 2:**
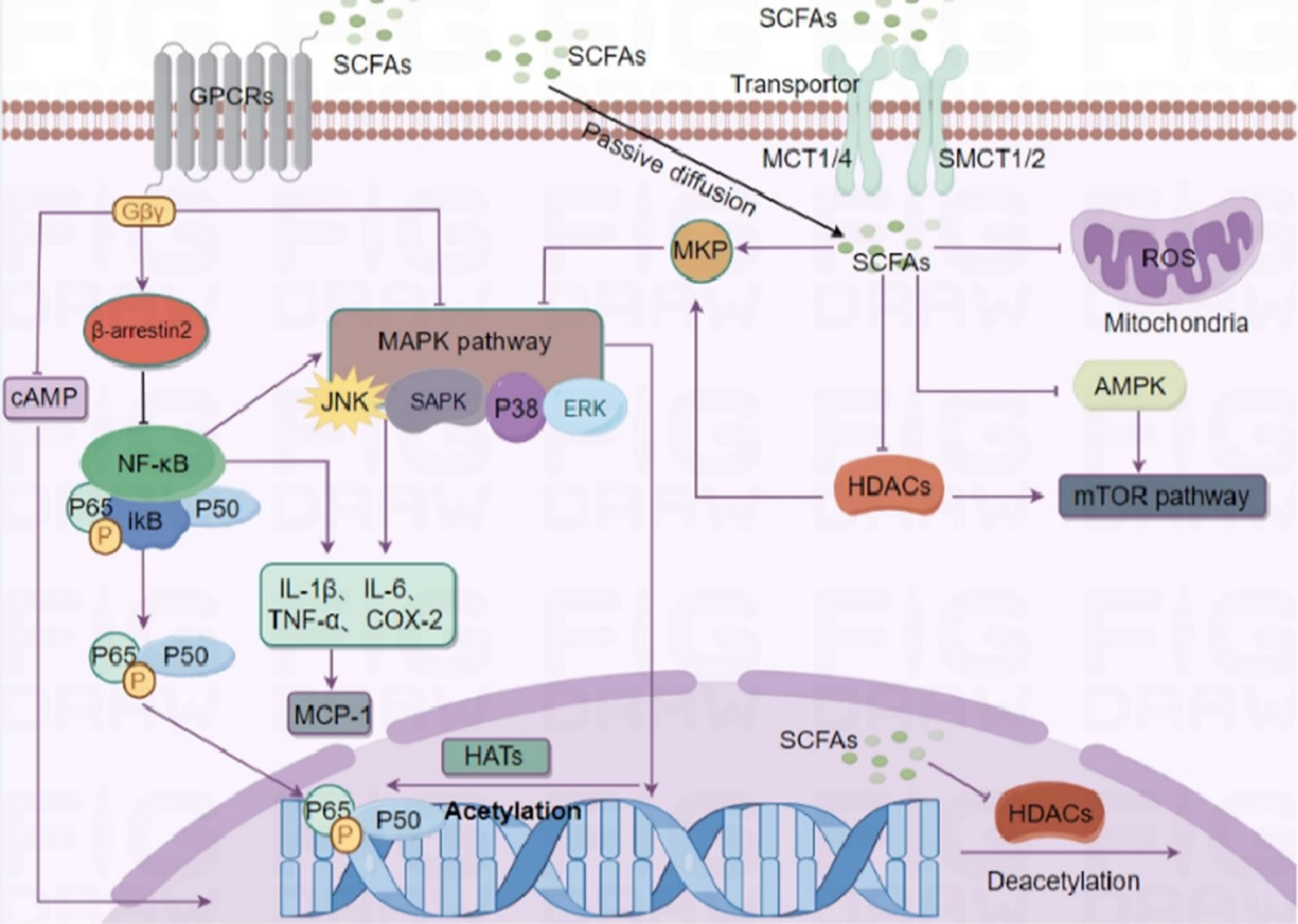
Mechanisms by which SCFAs regulate inflammatory responses. SCFAs regulate gene expression related to inflammation and immunity by inhibiting the NF-κ B and MAPK pathways and curtailing cAMP synthesis. Furthermore, they influence the mitochondrial/AMPK/mTOR pathway using transporters such as MCT1/4, SMCT1/2, and passive diffusion, leading to the inhibition of histone deacetylation. Additionally, they augment MKP expression, thus diminishing the MAPK pathway and resulting in anti-inflammatory effects

Persistent inflammatory response is considered to be an important component of CKD, which is often accompanied by elevated production and diminished elimination of pro-inflammatory factors, oxidative stress and acidosis, chronic and recurrent infections, and protein-energy wasting in CKD patients, contributing to the chronic inflammatory state of CKD by altering or interfering with renal microcirculatory regulation and perfusion distribution [39–41]. In CKD patients, the expression of pro-inflammatory cytokines such as IL-1, IL-6, TNF-α, and C-reactive protein tends to correlate negatively with renal function, which subsequently worsens the inflammatory condition and facilitates the onset of oxidative stress [42, 43]. It has been found that the inflammatory response in CKD patients directly or indirectly damages renal vascular endothelial cells, causes tubular injury and renal unit failure, and promotes the progression of CKD, which then activates the body’s defence mechanisms, resulting in the triggering of intracellular nuclear factor-κB (NF-κB), regulating the expression and secretion of a variety of renal tissue cells that express and secrete pro-inflammatory cytokines and chemokines, thus participating in inflammation and fibrosis in chronic kidney injury [44], in addition, the inflammatory response also reduces the production of erythropoietin (EPO) [45], decreases the sensitivity of erythrocytes to EPO [46], and interferes with iron metabolism [47] in CKD patients, thus further exacerbating their anaemic Status. Normally, NF-κB is silenced by binding to its inhibitor of NF-κB (iκB), However, when the p65 subunit of NF-κB is exposed, iκB will be degraded by polyubiquitination, thereby activating NF-κB and aggravate the inflammatory response. In contrast, SCFAs were able to significantly reduce NF-κBp65 subunit expression and increase iκB expression in renal epithelial cells, thereby inhibiting NF-κB activity, resulting in a decrease the expression of pro-inflammatory cytokines and chemokines, including IL-1β, IL-6, TNF-α, and COX-2. Furthermore, SCFAs mitigated the TNF-α-induced expression of monocyte chemotactic protein-1 (MCP-1), a potential inducer of fibrosis, thereby decelerating the progression of renal fibrosis [48]. In Colo 320 DM cells, all three primary SCFAs demonstrated a substantial suppression of NF-κB activity, with butyrate displaying the highest potency, followed by propionate and then acetate [49]. In addition SCFAs can penetrate the cell via transporters MCT-1、MCT-4 、SMCT-1and SMCT-2, which on the one hand can inhibit mitochondria, reduce ROS production and exert anti-oxidative stress functions, while inhibiting AMPK and mTOR pathways, inhibiting MAPK pathway by promoting MKP expression and enhancing histone acetylation by inhibiting HDACs. Regarding the mechanism by which SCFAs attenuate the inflammatory response in CKD patients as shown in Fig. 2. Kumar et al. [50] found that intervention of *Npr1*^±^ mice with treated sodium butyrate enhanced STAT1 acetylation in *Npr1* ± mice by inhibiting the HDACs pathway, and that acetylated STAT1 tended to bind more to the p65 subunit of NF-κB, increased iκB expression, and attenuated downstream pro-inflammatory factor expression and pro-fibrotic responses. SCFAs also inhibit the MAPK pathway through the negative regulator of G protein The negative regulator of signalling, GPR43-βarr2 (β-arrestin2), activates specific GPRs post-receptor phosphorylation and binds to βarr2 and blocks iκB phosphorylation and degradation [51], which effectively inhibits NF-κB activity and reduces inflammatory response expression [52]. In addition, increased NF-κB expression is always accompanied by activation of mitogenactivated protein kinase (MAPK), so inhibition of the MAPK signalling pathway is also one of the important ways for SCFAs to exert anti-inflammatory antioxidant effects. The MAPK signaling pathway primarily encompasses the following components: the c-Jun amino-terminal kinase (JNK)/stress-activated protein kinase (SAPK) pathway, the p38MAPK pathway, and the extracellular signal-regulated kinase (ERK), all of which play critical roles in the inflammatory response linked to ischemia–reperfusion injury [53]. Propionate was found to significantly reduce TNF-α-induced phosphorylation of p38 MAPK and JNK in renal cortical epithelial cells by binding to GPR41/43, which in turn attenuates fibrosis and inflammatory responses in CKD [53]. SCFAs also enhance the acetylation state of mitogen-activated protein kinase phosphatase 1 (mMKP-1) by inhibiting the HDACs pathway, and followed by promoting JNK and p38MAPK dephosphorylation, reversing MAPK signalling, and ultimately inhibiting LPS-induced expression of TNF-α, IL-1 and nitrite synthesis and reducing renal tubular cell apoptosis [54]. In studies on mice with ischaemic nephropathy, SCFAs can inhibit pro-inflammatory cytokine production, oxidative stress and apoptosis by inhibiting HDAC [55].

It has also been found that SCFAs can directly penetrate immune cells through either passive diffusion or the involvement of transporter proteins (MCT1/4 and SMCT1/2), exerting anti-inflammatory effects by modulating immune function [56]. SCFAs can inhibit inflammatory responses by decreasing immune cell migration, proliferation, recruitment, and cytokine levels, as well as inducing apoptosis [57], As shown in Table 2.

**Table 2:** SCFAs regulate immune imbalance in CKD patients through immune pathways

	Targeted cells	Ligand	Function	References
Anti-inflammatory	T-lymphocyte	Acetate; Propionate; Butyrate	Th1 cell↑; Th17 cell↑; Treg cell↑; IL-18↑	[58]
	LPS-induced monocytes	Acetate; Propionate; Butyrate	TNF↓; NF-κB↓; NOS↓	[59]
	Dendritic cell; Macrophage	Acetate; Propionate; Butyrate	IL-6↓; IL-12↓	[56]
	Macrophage	Butyrate	NOS↓; TNF↓; MCP-1↓; IL-6↓	[60]
	Endothelial cell	Butyrate	IL-6↓; ROS↓	[61]

### Inhibition of oxidative stress

Oxidative stress is a prominent characteristic of CKD and serves as a catalyst for disease progression. As CKD is in a chronic inflammatory state, mitochondrial dysfunction, cellular damage and apoptosis in damaged cells induce an increase in ROS production and exceed the antioxidant defences, leading to ROS accumulation in the body, triggering a downstream inflammatory cascade [62], activating the transcription factors NF-κB, AP-1, and p53, and promoting pro-inflammatory cytokine and chemokine production [63–65], further exacerbating the inflammatory state and promoting the disease progression of CKD [66] and renal fibrosis [67]. On the other hand, in CKD patients, the significant impairment of their antioxidant system results from the overproduction of reactive oxygen species (ROS), which reduces the activation of Nrf 2 (Erythroid-related nuclear factor 2). Nrf 2 is a cellular defense factor responsible for encoding antioxidants, and its reduced activation has been observed to directly hinder the expression of pro-inflammatory NF-κB target genes [68], which subsequently exerts a protective effect by regulating uremic inflammation and enhancing antioxidant defenses, whereas the overproduction of ROS in the course of CKD reduces the activation of Nrf 2, which further exacerbates renal fibrosis, tubular injury and hypoxia in CKD patients [69]. Normally, Nrf2 is typically sequestered in a quiescent condition by the cytoplasmic repressor Keap1 (Kelch-like ECH-associated protein 1), which is capable of facilitating rapid proteasomal degradation. However, under conditions of oxidative and electrophilic stress, Keap1 functions as an electrophilic sensor and liberates Nrf2. In this scenario, Nrf2 interacts with the small Maf protein (sMAF), which binds to phase II and antioxidant response elements (AREs) within the promoter regions of antioxidant enzyme genes, thereby countering oxidative stress [70]. SCFAs regulate different cellular oxidoreductase activities and P300-mediated nuclear factor E2-related factor by binding to GPR43 or acting as HDAC inhibitors 2 transcriptional activation to reduce ROS production, thus exerting an anti-oxidative stress effect [71]. Butyrate and propionate can maintain redox homeostasis by modulating Keap1-Nrf 2-dependent cell signalling pathways. Butyrate induces activation of nuclear factor Nrf 2 through recognition of GPR 109 A receptor and promotes ROS inactivation, thereby enhancing antioxidant defence protection [72]. Additionally butyrate, through HDAC inactivation, increases the production of histone HEK9ac, which induces epigenetic modifications on the Nrf 2 promoter and acts synergistically on Nrf 2 activation [73–77]. In CKD patients undergoing maintenance hemodialysis, the administration of propionate results in a decrease in the concentration of oxidative stress markers, including malondialdehyde, and enhances glutathione peroxidase activity. This results in a decrease in the overall concentration of oxygen free radicals and a reduction in oxidative stress levels [78].

### Modulation of autophagy

Autophagy is a cellular process responsible for recycling and removing damaged organelles, utilizing lysosomal degradation and disposing of intracellular wastes under the regulation of autophagy related gene (Atg), which is of significant importance in maintaining cellular homeostasis, influencing the aging process, modulating immunity, and preventing cellular damage. Autophagy is initiated in glomerular thylakoid cells, tubular cells, and podocytes under stressful conditions, such as hypoxia, nutritional deficiencies, infections, DNA damage, and oxidative stress, and autophagy assuming a pivotal role in the onset and progression of renal diseases [79]. The mammalian target of rapamycin, mTOR, pathway is responsive to environmental alterations and orchestrates numerous intracellular processes accordingly, and is one of the classical autophagy regulatory pathways involved in renal tubulointerstitial fibrosis [80]. mTOR pathway consists mainly of two mTORC 1 and mTORC 2 complexes, of which mTORC 1 contains a specific protein Raptor, whose activation involves phosphatidylinositol-3 kinase (PI 3 K) and the Akt pathway or AMPK signalling pathway [81], and is mainly involved in anabolism and inhibits autophagy. Amidst periods of stress and energy deficiency AMPK is activated and inhibits mTOR, facilitating the formation of autophagosomes [82]. Under conditions such as starvation and hypoxia, mTORC 1 is inhibited, which activates autophagy to remove cytoplasmic proteins from damaged organelles [83], which promotes the regeneration and repair of renal tubular cells, reduces the level of proteinuria, decreases glomerulosclerosis and podocyte loss, and slows down the advancement of CKD [84]. SCFAs cause the formation of autophagosomes through the inhibition of HDAC in T-cells, leading to the acetylation of p70S6K and phosphorylation of rS6 thereby modulating the mTOR signalling pathway [85] and promoting the production of T helper cell type 17 (Th 17), Th 1 and IL-10^+^ T cells. Supplementation with SCFAs decreased HDAC4 expression and increased histone acetylation, enhanced autophagy, autophagic vesicles with double membrane structure increased significantly in renal tissues, p-AMPK/AMPK protein ratio increased while p-mTOR/mTOR protein ratio decreased, renal lesions were significantly reduced, and pedunculated cell damage and proteinuria were significantly ameliorated [86], thus SCFAs may activate autophagy via the AMPK/mTOR signalling pathway and delay DN progression. Andrade-Oliveira et al. [55] found that SCFAs could achieve nephroprotective effects by promoting the expression of autophagy-related gene-7 (ATG-7) within renal tubular epithelial cells and increased levels of mitochondrial DNA in renal tissues, activating autophagy and inhibiting apoptosis. Studies have shown [85] that butyrate may potentially yield advantageous effects in cancers such as colon cancer through the activation of autophagy, achieved by inhibiting the mTOR pathway, and propionate may also induce autophagy by downregulating the mTOR signaling pathway [87]. A study by Pant [88] demonstrated that butyrate stimulates the generation of ROS, which then in turn inhibits Akt phosphorylation resulting in the suppression of mTOR and thereby leading to autophagy activation. The above findings only suggest that the activation of cellular autophagy by SCFAs exerts a renal protective effect, but the specific aspects of the regulation of cellular autophagy by SCFAs as well as the regulatory mechanisms are not yet fully understood, and need to be further investigated.

### Improvement of energy metabolism

SCFAs also act through energy metabolism in CKD. In CKD patients, insulin resistance can occur due to small molecule uremic toxins in the body or due to CKD complications including disrupted mineral metabolism and acid–base balance irregularities [89]. In contrast, Marzocco et al. [78] showed that in CKD patients on maintenance haemodialysis, sodium propionate reduced their plasma insulin levels and increased insulin sensitivity, but did not reduce their blood glucose levels.

**Fig. 3:**
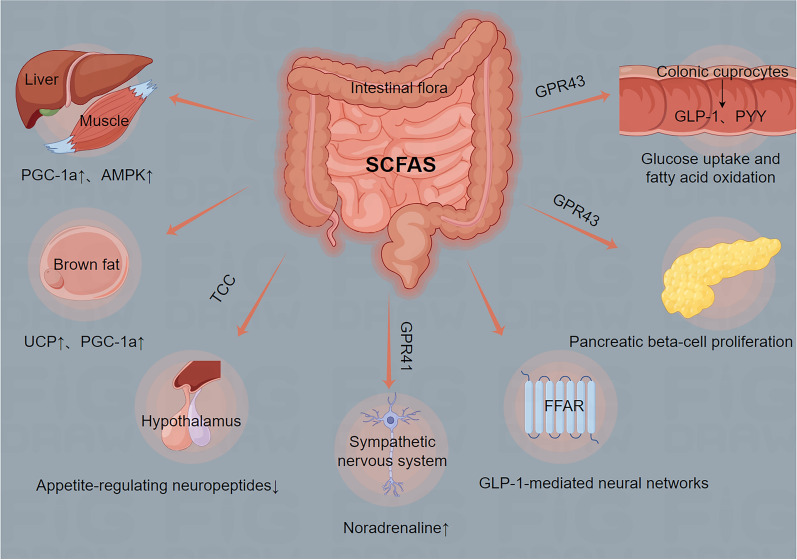
SCFAs improve energy metabolism pathways. SCFAs can enhance energy metabolism in CKD patients by eliciting the secretion of GLP-1 and PYY from colonic enteroendocrine cells, activating PGC-1a and AMPK through interactions with peroxisome proliferator receptors in muscle and liver, stimulating the expression of UCP and PGC-1a in brown adipose tissue, engaging GLP-1-mediated neural circuits via free fatty acid receptors, down-regulating the expression of appetite-regulating neuropeptides in the thalamus through the TCC activation, directly promoting the proliferation of pancreatic β-cells via GPR43 binding, and inducing the release of norepinephrine through GPR41 binding in the sympathetic nervous system

Propionate can stimulate an increase in insulin secretion and glucagon-like peptide-1 secretion by improving pancreatic β-cell function, which subsequently reduces blood glucose concentration and improves glucose homeostasis and insulin resistance [90]. The contribution of SCFAs to the regulation of glucose homeostasis may be attributed to their involvement in glucose synthesis [56]. The specific mechanisms are shown in Fig. 3 [56, 91–98].

### Immune pathways

Patients with CKD induce alterations in the intestinal immune system due to disturbances in the intestinal flora and the impairment of the intestinal barrier capacity, causing systemic immune imbalances and consequently immune-mediated nephropathy [99]. In addition, McDermott et al. [100] found that SCFAs directly modulate the immune system by promoting IL-22, Reg3γ, IgA, and IL-17 responses. SCFAs, especially butyrate, are able to bind to GPR109A on colonic epithelial cells, macrophages and dendritic cells, causing interleukin-10 (IL-10) secretion, reducing MCP-1 expression and vascular cell adhesion molecule-1 (VCAM1) release, and thus participating in the regulation of macrophage recruitment to and migration from the intestine [101]. SCFAs can also act on immune cells, regulating leukocyte transport by reducing the expression of chemokines in dendritic cells derived from human monocytes to inhibit macrophage and dendritic cell recruitment [102]. Mouse dendritic cells treated with propionate showed reduced expression of CD40, PD-L2 and CD86, inhibiting the immune response involving Th2 cells [103]. In neutrophils, butyrate inhibits neutrophil chemotaxis in vivo by decreasing the expression of chemotactic receptors C5AR and CXCR 2 through binding to GPR43 [104] and inhibits the production of the pro-inflammatory cytokine TNFα in neutrophils [105], modulating their production of reactive oxygen species and phagocytosis [106]. SCFAs also act on HDACs by inhibiting the action of monocytes and neutrophils, reducing TNF production and leading to inactivation of transcription factor-nuclear factor-κB (NF-κB) [107], attenuating the inflammatory response in CKD patients. Yao et al. [56] found that SCFAs can not only participate in the regulation of the immune system by acting on innate immune cells (e.g., macrophages, neutrophils, and dendritic cells), but also regulate the differentiation of T and B cells and the antigen-specific adaptive immunity they mediate. Propionate and acetate can activate regulatory T lymphocytes by binding to GPR 43 inducing the transcription factor forkhead box P3 (FOXP 3), leading to cell expansion and differentiation into mast cells, neutrophils, and eosinophils, respectively, and are involved in the regulation of the development and differentiation of Treg cells, leading to intestinal immunity [108]. Park et al. [18] found that SCFAs, as inhibitors of HDACs, were able to promote the differentiation of T cells into effector T cells and Treg cells, as well as to enhance mTOR-S6K activity, which is required for T cell differentiation. Yang et al. [109] showed that SCFAs were able to promote the development of mouse splenic CD4^+^ T cells and interleukin-22 (IL-22) production by innate lymphocytes. Meyer et al. [110] detected an augmentation in the quantity of regulatory T cells in peripheral blood following propionate supplementation in haemodialysis patients. Yang et al. [111] demonstrated that Th17 cells are a key contributor to the development of intestinal inflammation resulting from renal ischemia/reperfusion injury and this was accompanied by a decrease in the amount of SCFAs in the faeces, speculating that there may be a link between the two. SCFAs are essential in governing both regulatory and effector T cells, potentially influencing the immune milieu and gene expression regulation [56]. As for B cells, SCFAs can inhibit histone deacetylation in B cells to regulate the differentiation of B cells into plasma cells to produce more antibodies [112], in addition, SCFAs can increase the metabolism of B cells, such as adipogenesis, oxidative phosphorylation, and glycolysis and provide energy for plasma cell differentiation [113]. In addition, SCFAs can regulate immune responses through adipocytes [56]. Butyrate in SCFAs can significantly increase the expression of MCP-1 in cells after interacting with free fatty acid receptor 3 (FFAR 3) in preadipocytes, thus participating in the regulation of immune response [114]. SCFAs can also stimulate inflammatory vesicles by binding to GPR43 on intestinal mucosal epithelial cells (IECs), thus activating interleukin-18 (IL-18) downstream signaling, which is involved in repairing and maintaining the integrity of the intestinal epithelium and reducing the absorption of uremic toxins into the bloodstream in CKD patients [115]. Butyrate, an inhibitor of HDACs, modulates the expression of transforming growth factor β in intestinal mucosal epithelial cells (IECs), thereby inducing their differentiation into Treg cells [116].

## SCFAs and CKD complications

### Vascular calcification

Vascular calcification, which is the most important factor influencing the complication of cardiovascular diseases and death in CKD patients, is a complex and highly regulated biological process [117], which is mainly manifested by the accumulation of Ca^2+^ and phosphate in the intima and mid-membrane of the vessels. VC can be divided into two main pathological patterns: media calcification and intimal calcification [118]. The former mostly occurs in the early stage of CKD without inflammation or lipid abnormalities and is prone to cause cardiac dysfunction [119], while the latter is mostly composed of macrophages and lipid substances deposited in the intima of blood vessels in the middle and late stages of CKD, which may induce coronary ischemia [120]. In CKD patients, due to the presence of disturbances in the metabolism of minerals such as calcium and phosphorus, oxidative stress, inflammatory responses and endocrine imbalances, vascular calcification mediated by a combination of the osteoblast phenotype and cells synthesizing mineralization-regulating proteins occurs in the vascular smooth muscle cells, which converts the VSMC cell phenotype from constrictor to osteoclast, and at the same time, calcium (Ca^2+^) and phosphate accumulate in the meso- and intima-media of the vessels, forming vascular calcification [117]. The prevalence of VC is as high as 65% in the chronic renal insufficiency cohort (CRIC) [121] and 74% in the end-stage renal disease (ESRD) population [122]. Studies have shown that CKD dialysis patients are 8 times more likely to develop vascular calcification than the normal population, up to 90% [123], and the onset of VC in CKD patients occurs more than 10 years earlier than in normal subjects [124]. VC exacerbates CKD and the incidence of CVD, so early and aggressive prevention and treatment of VC is essential for controlling the disease and improving quality of life.

**Fig. 4:**
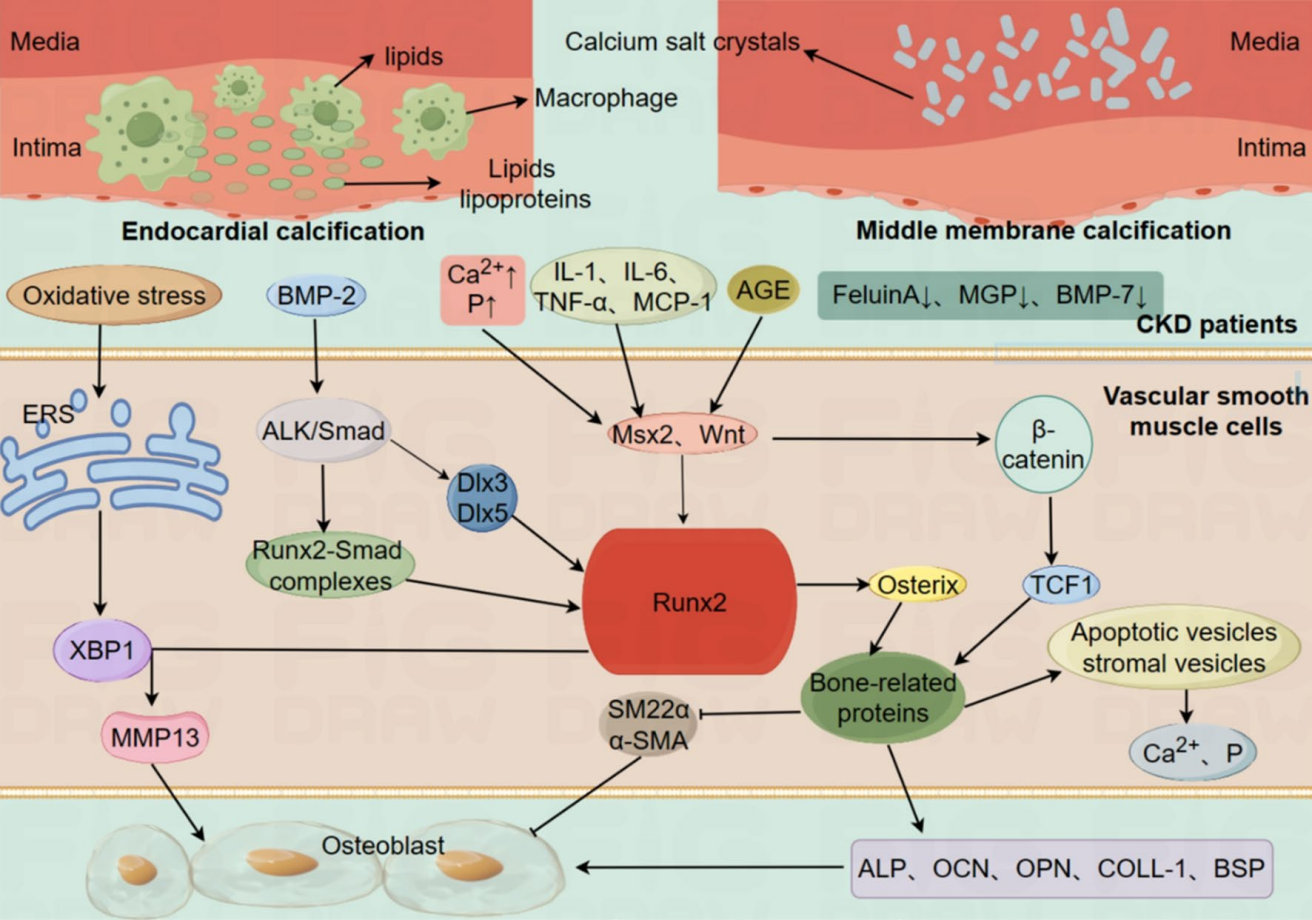
Characteristics and formation mechanisms of vascular calcification. In the face of environmental changes (e.g. oxidative stress, disturbed calcium and phosphorus metabolism, increased release of pro-inflammatory factors, etc.), VSMCs activate the ALK/Smad pathway, Msx2, and Wnt pathway to promote the transformation of the VSMC cell phenotype from contractile to osteoblastic

Elevated blood phosphorus in CKD patients enters vascular smooth muscle cells (VSMCs) via Phosphate transporter-1 (Pit-1) [125]. Up-regulating the expression of osteogenic markers such as Runt-related transcription factor 2 (Runx2) and bone morphogenetic protein2 (BMP-2). Moreover, it down-regulates the expression of contractile markers such as smooth muscle22α (SM22α) and α-smooth muscle actin (α-SMA), induces VSMCs phenotype transformation from contractile type to osteoblasts [126] or triggers cell apoptosis. Loss of its ability to synthesize calcium inhibitors is accompanied by the release of extracellular vesicles that promote calcium aggregation and the formation of calcium crystals. Moreover, inflammatory cytokines in CKD patients can promote the expression of Pit-1 and accelerate the entry of calcium phosphate into VSMCs [127]. The elevation of parathyroid hormone (PTH) can cause the increase of blood calcium concentration, promote VSMCs apoptosis and inhibit autophagy [128], and then promote the secretion of matrix vesicles [129], leading to calcification. Oxidative stress in CKD patients Oxidative free radicals can activate Endoplasmic Reticulum stress (ERS) and increase the expression of transcription factor X-box binding protein 1 (XBP1). Binding Runx2 promoter can increase the expression of matrix metalloproteinase 13 (MMP13), and then initiate and promote VSMCs calcification, while reducing the generation of oxygen free radicals can effectively delay calcification [130]. As a member of the transforming growth factor-β (TGF-β) superfamily, BMP-2 binds to its receptor to promote Runx2-Smad complex formation and up-regulate Runx2 expression through activation of the ALK/Smad pathway. In addition, BMP-2 can activate the osteogenesis-related transcription factor Osterix to promote osteoblast differentiation [131]. At the same time, BMP-2 can also activate the upstream regulatory factors of Runx2 homeobox transcription factor 3(Dlx3) and Dlx5, thereby mediating the transcription regulation pathway of osteoblast differentiation and VC [132]. In CKD mice, BMP2 overexpression accelerated calcification, while BMP2 inhibition reduced calcification. In CKD patients, the levels of protective agents such as fetuin-A, γ-Matrix Gla Protein (MGP) and pyrophosphate, which protect blood vessels from calcium and phosphorus and inhibit VC, are decreased. As the main component of vascular intima, ECs are transformed and produce a variety of active substances under stimulating factors such as inflammation in CKD patients, as well as release vesicles, which promote intercellular communication and lead to VC [133]. The specific mechanisms by which vascular calcification occurs in CKD patients are shown in Fig. 4 [128, 134–137].

**Fig. 5:**
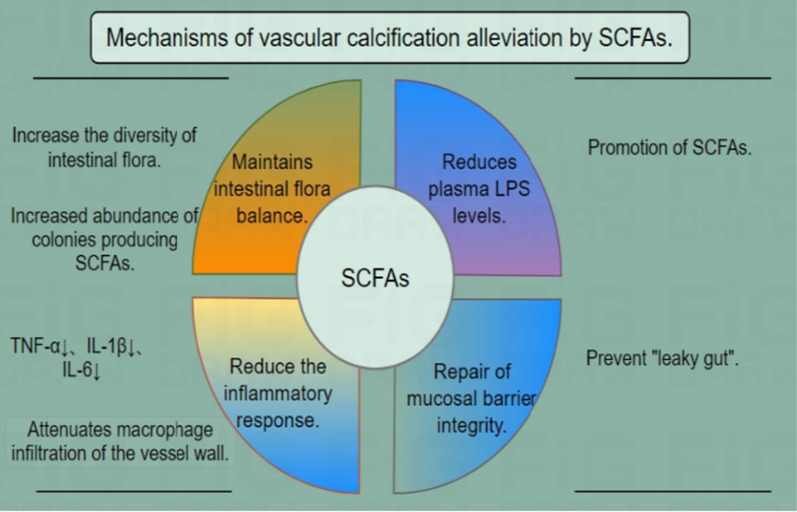
Mechanisms by which SCFAs alleviate vascular calcification. In patients experiencing vascular calcification, there is a tendency for the intestinal microbiota to experience homeostatic imbalance, characterized by decreased SCFA production, elevated LPS content, compromised mucosal barrier integrity, and intestinal “leakage”. This phenomenon can initiate a systemic inflammatory response and, in turn, worsen vascular calcification. And SCFAs may attenuate the occurrence of CKD complicated by vascular calcification by reducing these homeostatic imbalances

Jianlong Yan [138] et al. performed Spearman's correlation analysis of blood and feces from 92 patients, and the results showed that SCFAs, especially propionate, were independent protective factors inhibiting vascular calcification, and that the α-diversity of the intestinal flora in rats with vascular calcification was significantly decreased, and the intestinal flora was dominated by Bacteroidetes, Firmicutes and Proteobacteria, whereas oral administration of propionate reversed these trends and increased the α-diversity of the intestinal flora and the abundance of intestinal flora producing SCFAs. Propionate can greatly reduce plasma endotoxin (LPS) levels and promote the production of SCFAs, reduce plasma concentrations of inflammatory factors such as TNF-α, IL-1β and IL-6, attenuate macrophage infiltration in the vessel wall, re-establish a normal microbial community, and attenuate inflammatory responses to improve vascular calcification. In patients with vascular calcification, the intestinal flora tends to be homeostatically imbalanced, with decreased SCFA production, increased LPS content, impaired mucosal barrier integrity, and intestinal “leakage”, which can trigger a systemic inflammatory response and ultimately exacerbate vascular calcification. Propionate supplementation and fecal flora transplantation maintains the balance of intestinal flora, promotes the production of SCFAs, and reduces LPS levels. In this way, propionate protects the integrity of the mucosal barrier, prevents intestinal “leakage”, inhibits the inflammatory response, and thus alleviates vascular calcification. In contrast, it was found [139] that butyrate significantly accelerated high phosphorus-induced calcification of VSMCs and aortic rings, as well as vitamin D3 (vD3)-induced vascular calcification in mice. Butyrate upregulated the expression of the osteogenic genes Runx2, Msx2 protein levels partly through inhibition of histone deacetylase (HDAC) and Gpr41- and Gpr109a-mediated NFκB signaling, which led to an increase in the expression of its target gene, Alp, in vascular smooth muscle cells, and the subsequent degradation of PPi pyrophosphate, a key inhibitor of vascular calcification [140], and decreases the level of the vascular calcification protective factor Opg protein, which promotes calcification and osteogenic differentiation in VSMCs. Figure 5 illustrates the mechanism through which SCFAs mitigate vascular calcification.

### High blood pressure

Within the study of the Chronic Renal Insufficiency Cohort (CRIC), 67–92% of patients were associated with hypertension [141]. In addition, the prevalence of hypertension increases progressively as renal disease progresses and renal function declines, while control rates are low [142, 143]. In contrast, SCFAs, mainly propionate and butyrate, have been found to interact with G protein-coupled receptors (Gpr41, Gpr43) and olfactory receptor 78 (Olfr78) thereby affecting host blood pressure [144]. Propionate has a higher affinity for Gpr41 than Olfr78, so low doses of propionate cause a decrease in blood pressure by activating Gpr41, promoting inositol 1,4,5-trisphosphate production, inducing elevated intracellular calcium ions, ERK1/2 activation, and inhibiting cAMP accumulation [145], whereas high doses of propionate moderately increase blood pressure by activating Olfr78 to antagonise the hypotensive effects of the antihypertensive effect of propionate to prevent large fluctuations in blood pressure due to normal physiological changes in SCAFs concentration. Butyrate is able to activate the podocyte GPR109A on the glomerular basement membrane to attenuate glomerulosclerosis and tissue inflammation to ameliorate proteinuria and lower blood pressure [146]. SCFAs also exert a potent anti-inflammatory effect by regulating the activity of immune cells, thereby attenuating the damage to target organs caused by hypertension. Owing to the HDAC inhibitory properties of SCFAs, propionate can induce the differentiation of regulatory T cells (Treg) that express the transcription factor Foxp3. In contrast, butyrate not only encourages the direct differentiation of CD41^+^ T cell precursors into Treg cells but also enhances dendritic cells’ capacity to foster Treg cell differentiation [147], promoting Treg production and function, augments IL-10 secretion, and mitigates the systemic T cell response to AngII [148]. Propionate mitigates the indices of AngII-induced cardiac hypertrophy by inhibiting the elevation in mRNA expression of vascular cardiac brain natriuretic peptide (Nppb) and β-myosin heavy chain (Mhy7). Additionally, it prevents AngII-triggered escalation of interstitial and perivascular myocardial fibrosis. Beyond the vascular system, SCFAs sensing receptors, including Olfr78 and GPR-43, as well as GPR-41, are found in the sympathetic ganglia. This allows SCFAs to directly modulate the sympathetic nervous system by activating GPR41 through the Gpy- PLCp- MAPK signaling pathway. On the one hand, propionate can generate action potentials via GPR41 to promote positive feedback release of norepinephrine from sympathetic nerve endings, and on the other hand, the ketone body p-hydroxybutyrate produced during starvation or diabetes inhibits sympathetic nervous system activity by antagonising SCFA-GPR41 [19]. SCFAs may also be activated via the reductio coenzyme II oxidase -intracellular reactive oxygen species pathway interfering with gut-neuron communication in the paraventricular nucleus of the hypothalamus or directly reducing norepinephrine production thereby inhibiting sympathetic overactivation [149].

### Other complications

The course of CKD is complex, and in addition to disorders of calcium and phosphorus metabolism and hypertension, patients with CKD are often accompanied by abnormal lipid metabolism. Abnormal accumulation of lipids and metabolic disorders have been found in the renal tissues of CKD patients [150], and accumulation of lipids in the kidney results in podocyte dysfunction and apoptosis [151], glomerulosclerosis, and tubulointerstitial damage, which then further aggravate the course of CKD [152]. Whereas SCFAs, especially acetate, play a role in cholesterol synthesis and the generation of new fat, both processes can be inhibited by propionate [153]. Consequently, the acetate-to-propionate ratio may be a critical factor in determining which SCFAs are involved in the regulation of lipid metabolism. In addition, studies have shown that propionate, when administered independently, leads to a reduction in visceral and hepatic fat [154]. In a study by Yu et al. [155], it was demonstrated that supplementing with butyrate elevated the expression of SESN2 and CRTC2 genes in the mouse liver, subsequently reversing endotoxemia, thereby improving the body's lipid metabolism, reducing fat intake and increasing the ability of cells to use energy to generate calories. This may be related to the fact that SCFAs bind to GRP43 acting on white adipose tissue and brown adipose tissue to inhibit fat accumulation and promote mitochondrial biogenesis, respectively [96]. Individuals with CKD frequently experience a decrease in erythropoietin (EPO) secretion, and in addition, the inflammatory state of CKD affects the body's iron status, with ferritin increasing in response to inflammatory levels [156], leading to erythropoietin resistance [157], which then further exacerbates the anaemic state of the organism. Supplementation of SCFAs in CKD patients was found to result in a decrease in body ferritin concentration and a significant increase in transferrin saturation [156], and an improvement in haemoglobin levels, which subsequently improves the anaemic state of the patients with CKD. CKD patients are prone to infections due to lowered immunity. And studies have shown that SCFAs exhibit to have anti-microbial properties. Fernandez-Rubio et al. [158] showed that butyrate can control Salmonella infections in the organism by disrupting osmotic pressure and pH balance, affecting microbial nutrient uptake and energy production, and other mechanisms. In a study by Hong et al. [159], it was illustrated that SCFAs can exert diverse inhibitory effects on oral microorganisms even at micromolar concentrations. Additionally propionate and hexanoic acid can exert anti-microbial activity by promoting the body to increase the expression of anti-microbial peptides [160].

## Hazards of SCFAs on kidney disease

However, not all findings emphasise the renoprotective role of SCFAs. The primary constituent of the olfactory signaling pathway, Olf78, is located within the kidney, where it assumes a pivotal functional role in the control of glomerular filtration rate (GFR) and the release of renin. Olfr78 interacts with SCAFs to activate Gα, which stimulates the production of cAMP in the paraglomerular cells, leading to the secretion of renin and the initiation of the renin–angiotensin–aldosterone system, which in turn increases blood pressure [161]. Park et al. [162] found that prolonged and slowly increasing the dose of oral SCFAs to higher than physiological levels resulted in the accumulation of effector T cells (Th1 and Th17) and regulatory T cells in the ureteral and renal tissues of mice, which induced T-cell-mediated ureteritis and consequently, hydronephrosis. In addition, mice given a high-fibre diet have elevated levels of intestinal butyrate, which increases the chance of E. coli infection, and elevated levels of Gb3 in the gut and kidney, leading to severe kidney damage [163]. In contrast, it was found [139] that butyrate notably accelerated high-phosphorus-induced calcification in VSMCs and aortic rings, as well as vitamin D3 (vD3)-induced vascular calcification in mice. Butyrate upregulated the expression of osteogenic genes such as Runx2 and Msx2 protein levels, in part, by inhibiting histone deacetylase (HDAC), along with Gpr41- and Gpr109a-mediated NF-κB signaling, which resulted in an increase in the expression of its target gene, Alp, in vascular smooth muscle cells, ultimately leading to the degradation of pyrophosphate (PPi), a crucial inhibitor of vascular calcification [140], and decreases the level of the vascular calcification protective factor Opg protein, which promotes calcification and osteogenic differentiation in VSMCs. In addition, SCFAs exacerbate the inflammatory response in CKD patients in vivo under certain conditions, and it has been shown that when SCFAs bind to GFR41/43, it further activates downstream mTOR, PI3K, or MAPK signalling pathways to exhibit a pro-inflammatory effect [164], and furthermore, SCFAs, especially acetate, are known to be effective in promoting inflammation by activating GFR41 or GFR43 and its downstream extracellular signal-regulated kinase 1/2 (ERK 1/2) signalling pathway and MAPK/p38 signalling pathway to up-regulate the production of pro-inflammatory cytokines and chemokines such as IL-6, CXCL 1 and CXCL 2. These studies suggest that SCFA also activate FFAR 2 and FFAR 3 to participate in pro-inflammatory effects. The possible mechanisms underlying the two entirely contrasting roles of SCFAs in inflammation still require further investigation. According to the current study, the different effects of SCFAs on the kidney were reported by Serino [165] to be associated with the concentration of SCFAs. This reflects the importance of SCFAs concentration assay in renal diseases and further studies are necessary to establish the pharmacological concentration.

## Strategies for regulating metabolites in the intestinal flora

A growing number of experiments are aimed at modifying the host gut microbiota and promoting the SCFAs synthesis pathway as a means of treating and preventing CKD. Considering the multifactorial nature of SCFAs production, several strategies can be employed to increase SCFAs levels in patients with CKD, such as altering dietary patterns, using probiotics and prebiotics to modulate the intestinal flora, and adsorbents to adsorb uremic toxins.

### Change of diet

Alterations in dietary patterns can induce changes in the gut microbiota composition within as little as 1 day. As a precursor for the synthesis of SCFAs by the gut flora, the intake of foods with dietary fibre and resistant starch can increase the level of SCFAs and delay the development of CKD [166]. High dietary fibre dietary intake was found to reduce the risk of inflammatory states and morbidity and mortality in CKD patients, with the correlation being stronger with higher amounts of dietary fibre consumed [167]. Sanchezetal et al. found that supplementing dietary fiber to organisms on a high-protein and high-fat diet elevated the abundance of beneficial microorganisms, lowered the concentration of harmful microbial metabolites, and heightened the production of SCFAs [168].

A low-protein diet is the most common dietary intervention for CKD patients. The results of the gut microbiota meta-analysis revealed substantial alterations in the gut microbiota of individuals following a low-protein diet compared to those on a normal protein diet, primarily characterized by the proliferation of SCFAs-producing *Mycobacteriaceae* [169] Thus the increased production of SCFAs in the low-protein dietary pattern helps to slow down the renal burden and alleviate the course of CKD [170].

### Use probiotics, prebiotics

Probiotics are live microorganisms administered in appropriate amounts to the host to restore normalcy and health benefits to the host by modifying the composition of gut microorganisms, competing for nutrients and inhibiting bacterial colonization of attachment sites, and by interacting with the host and gastrointestinal microbiota to inhibit the proliferation of harmful bacteria [171]. Probiotics are a cost-effective, minimally invasive intervention with few side effects, typically comprising live bacterial strains such as *bifidobacteria*, *lactobacilli*, and *streptococci*. These probiotics are capable of exerting antimicrobial effects through the production of bacteriocins [172], enhancing intestinal barrier function to reduce the absorption of enterogenic uremic toxins in CKD patients [173], slowing down the proliferation of pathogenic bacteria, enhancing the catabolism of waste molecules, attenuating the inflammatory response through the blockade of receptors, and participating in immune responses. Consequently, they enhance intestinal barrier function and alleviate imbalances in the intestinal flora. Ultimately, they lead to a notable improvement in renal function [174]. Clinical studies have shown [175] that individuals with CKD stages 3 and 4 who were administered a Renadyl preparation containing *Lactobacillus acidophilus*, *Streptococcus thermophilus*, and *Bifidobacterium longum* for a duration of 6 months experienced a substantial decrease in urea nitrogen levels and an improvement in their quality of life. Zhu et al. [176] found that supplementation of CKD patients with Lactobacillus casei increased the level of serum SCFAs and subsequently improved the inflammatory response of local macrophages and renal tubular epithelial cells.

Prebiotics include almost or completely indigestible carbohydrates such as inulin, oligofructose, oligogalactose, oligosoybean sugar, and oligo xylose [177], promoting the proliferation of advantageous bacteria like Bifidobacterium and Lactobacillus within the gastrointestinal tract, while inhibiting other species of bacteria. Oral prebiotics were observed to considerably mitigate inflammation and oxidative stress in CKD, lower serum creatinine and urea nitrogen levels, and impede the progression of CKD by suppressing renal tubulointerstitial fibrosis. These prebiotics have been effectively employed in CKD hemodialysis patients [178]. By taking advantage of the synergistic effects of prebiotics and probiotics and making multiple combinations, which become Synbiotics, often with better efficacy, such as OAT fiber/*Lactobacillus* plantarum and FOS/L *sporotrichum* [179]. Synbiotics have been shown to improve intestinal ecological dysregulation, reduce indole production and absorption, and improve CKD progression in CKD rats [180].

### Adsorbents for uremic toxins

The combination of adsorption of uremic toxins and reduction of intestinal toxin absorption through oral administration of activated charcoal adsorbents or compound binding agents is beneficial in reducing complications in CKD patients. Clinical studies have demonstrated that activated charcoal klimet (AST-120), an oral activated charcoal adsorbent, significantly reduces uremic symptoms in patients with CKD, as well as reduces proteinuria and shortens the duration of dialysis, and significantly reduces serum p-cresol sulphate, which has been used to attenuate microbial alterations of the intestinal tract and systemic inflammation [181]. Nakamura et al. [182] found a reduction in IL-6 and serum creatinine levels, along with a significant improvement in proteinuria after applying AST-120 to 50 patients with CKD. As a polyphosphate-binding agent, Sevelamer binds uremic toxins, and studies have shown that CKD patients who took Sevelamer for 3 months had significantly lower C-reactive protein, glycated haemoglobin, and LDL levels, and significantly higher HDL levels compared to the control group (who did not take Sevelamer), suggesting that Sevelamer may ameliorate inflammation and dyslipidaemia and may have some therapeutic efficacy in CKD patients [183].

### Fecal microbial transplantation

Fecal microbial transplantation (FMT), which primarily involves the transfer of fecal microbial extracts from a healthy donor to a diseased recipient to restore its intact gut microbial community and function, is a recognized and valuable treatment for recurrent *Clostridioides* difficile infection (CDI) [184]. Nowadays, FMT has been widely used for the treatment of both intestinal and extra-intestinal diseases, such as metabolic diseases, neuropsychiatric diseases and autoimmune diseases [38]. It is widely used that the washed microbiota transplantation (WMT) technique reduces the incidence of FMT-related adverse events such as fever, diarrhea, abdominal pain, bloating, nausea, and vomiting without compromising its efficacy [185]. Although there have been a number of animal experiments observing the efficacy of FMT in kidney diseases. FMT reduces albuminuria and modulates renal phenotype in antibiotic-treated humanized IgAN mice [186]. However, whether FMT can be applied to clinical patients with renal diseases, this still needs to be cautious. In addition, numerous studies are still required to delve deeper into the FMT technique, such as the selection of donor, long-term stability of donor faeces, safety and treatment modalities.

### Other strategies

In addition to the above commonly used methods to improve the metabolism of gut flora, there are a number of other strategies to modulate the gut subsequently playing a significant role in the treatment of CKD. Physical exercise has been found to have the potential to reestablish the abundance and homeostasis of the gut flora by increasing the relative abundance of butyrate-producing bacteria and the ratio of Bacteroides fragilis (Bacillus fragilis) to the thick-walled phylum of bacteria, and butyrate levels in the gut [187], potentially improving the course of CKD. In recent years, genetic engineering techniques have also been applied to reduce regulated intestinal metabolites and ameliorate diseases. Oral administration of microencapsulated genetically engineered live Escherichia coli DH5 cells to CKD rats effectively reduced their levels of uremic toxins in vivo [188]. Devlin [189] found that deletion of a family of tryptase enzymes widely distributed in the intestinal commensal anaplastic bacillus flora eliminated the production of indole in vivo and greatly reduced the complications and hazards of CKD patients. Thus microbiota engineering technology, although not yet at the level of clinical application, is it may be a promising method for realizing precision medicine. As one of the most prevalent microorganisms in the human gut, *Bacteriophages* act as ecological antagonists of gut bacteria by preying on commensal bacteria present in the gut, thereby promoting the stability of bacterial communities [190]. Thus, *Bacteriophages* are able to accurately regulate the host microbiome, achieve inhibition of certain harmful microorganisms, improve intestinal homeostasis and promote its healthy state [191], thereby effectively slowing down the progression of chronic kidney disease (CKD).

## Conclusions

CKD remains a prominent global cause of mortality. The function of gut-derived SCFAs in kidney disease has become an exciting area in recent years, and the interactions between gut flora and SCFAs have multifaceted effects on chronic kidney disease, such as attenuating the inflammatory response in patients, inhibiting oxidative stress, modulating autophagy, and regulating energy metabolism and immune pathways. While the current treatment of CKD is focused on slowing disease progression and protecting renal function, understanding the link between gut flora and CKD is expected to open new research horizons for the future management of kidney disease, provide an opportunity to identify SCFAs as a new target for the treatment of chronic kidney disease, and pave the way for future preventive and therapeutic approaches. Numerous studies have employed dietary interventions, probiotics, FMT, genetic engineering and other technologies, etc. to modulate gut microbiota diversity and SCFA levels in patients, thereby enhancing the gut microbiome, elevating SCFA concentrations, and mitigating CKD risk. However, given the complex composition of gut microbiota, the whole field of SCFA in kidney disease is still in its infancy. Although encouraging results have been achieved in recent years for related studies, concerns remain about the safety of gut microbiome modulation strategies, and most studies have been limited to cellular and animal experiments without sufficient reliable clinical application effectiveness studies. Thus, further investigation is warranted to comprehensively comprehend the specific mechanisms of action as well as develop optimal application strategies. Consequently, longitudinal human studies are imperative in elucidating the roles of gut microbiota and SCFAs in kidney disease while facilitating advancements in therapeutic strategies.

## Data Availability

Not applicable.

## Abbreviations

BMP-2: Bone morphogenetic protein
AGE: End-stage glycosylation end-products
FeluinA: Fetal globulin A
TCF1: Transcription factor T-cell factor 1
XBP1: Transcription factor X box binding protein
OCN: Osteocalcin
BMP-7: Bone-forming protein-7
ERS: Endoplasmic reticulum stress
MMP13: Collagen X matrix metalloproteinase 13
COLL-1: Collagen-1
MGP: Matrix Gla protein
ALP: Alkaline phosphatase
Dlx3、Dlx5: Homology box transcription factors 3, 5
BSP: Osteoprotegerin
Osterix: Osteogenesis-related transcription factors
OPN: Bridging protein
